# Role of Telomerase in the Cardiovascular System

**DOI:** 10.3390/genes7060029

**Published:** 2016-06-17

**Authors:** Mark Zurek, Joachim Altschmied, Stefanie Kohlgrüber, Niloofar Ale-Agha, Judith Haendeler

**Affiliations:** 1 IUF-Leibniz Research Institute for Environmental Medicine, 40225 Duesseldorf, Germany; mark.zurek@uni-duesseldorf.de (M.Z.); joachim.altschmied@uni-duesseldorf.de (J.A.); stefanie@gonnissen.de (S.K.); niloofar.ale-agha@uni-duesseldorf.de (N.A.-A.); 2 Central Institute of Clinical Chemistry and Laboratory Medicine, Medical Faculty, University of Duesseldorf, 40225 Duesseldorf, Germany

**Keywords:** Telomerase Reverse Transcriptase, cardiovascular cells, aging

## Abstract

Aging is one major risk factor for the incidence of cardiovascular diseases and the development of atherosclerosis. One important enzyme known to be involved in aging processes is Telomerase Reverse Transcriptase (TERT). After the discovery of the enzyme in humans, TERT had initially only been attributed to germ line cells, stem cells and cancer cells. However, over the last few years it has become clear that TERT is also active in cells of the cardiovascular system including cardiac myocytes, endothelial cells, smooth muscle cells and fibroblasts. Interference with the activity of this enzyme greatly contributes to cardiovascular diseases. This review will summarize the findings on the role of TERT in cardiovascular cells. Moreover, recent findings concerning TERT in different mouse models with respect to cardiovascular diseases will be described. Finally, the extranuclear functions of TERT will be covered within this review.

## 1. Introduction

The ends of chromosomes are capped with telomeres, which protect the chromosomes from end-to-end fusion. With each cell division those telomeres—noncoding double-stranded repeats of G-rich tandem DNA sequences (TTAGGG in vertebrates)—are shortened because the DNA polymerase protein complex cannot completely replicate the sequences present at the ends of the chromosomes. Due to this end replication problem those telomeres are shortened during each cell division. This process is counteracted by the enzyme Telomerase. The Telomerase holoenzyme consists of the catalytic subunit Telomerase Reverse Transcriptase (TERT) and the non-coding Telomerase RNA Component (TERC), which serves as the template for the elongation of the telomere sequences by TERT [1]. From textbooks it is known that Telomerase is active in tumor cells and stem cells of highly regenerative tissues and the infinite life span of those cells has been clearly attributed to highly active Telomerase. Forcing cells to divide in cell culture leads to reduced Telomerase activity and to replicative senescence after a predictable number of cell divisions [2]. The role of TERT in this process has been causally proven, because ectopic expression of TERT is widely used to immortalize human cells [3].

**Figure 1:**
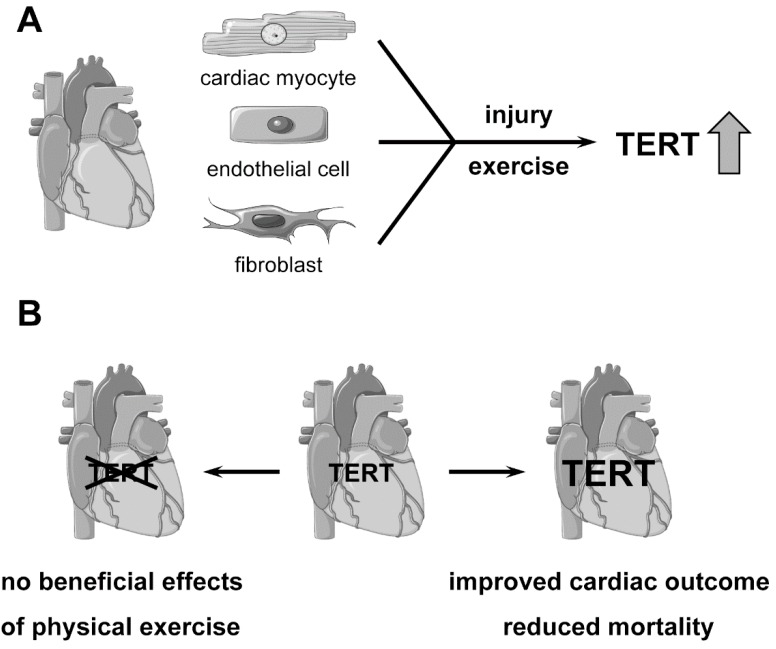
Regulation and functions of TERT in the heart. (**A**) TERT is present in cardiac myocytes, endothelial cells and fibroblasts of the adult heart. Heart injury as well as physical exercise increase TERT expression and Telomerase activity; (**B**) TERT deficiency completely blunts the protective effects of voluntary running in the heart. Increasing TERT levels in the heart using transgenic or viral approaches improves cardiac outcome and significantly reduces mortality.

It has been proposed for a long time that Telomerase activity is absent from human somatic cells [4]. However, there is accumulating evidence that substantial Telomerase activity is present in differentiated, non-dividing somatic cells of the cardiovascular system (Figure 1A) [5,6,7,8,9]. This is of particular importance since cardiovascular diseases (CVD) are still the leading cause of death worldwide. CVD include e.g., inter alia, chronic heart failure, coronary artery disease and myocardial infarction. All of these diseases have a primary defect in the heart or in the blood vessels, and there is emerging evidence that Telomerase has a protective effect against CVD.

Therefore, this review will focus on the role of Telomerase and in particular of TERT in the heart and in the vessels. Understanding this enzymes’ functions in these tissues could, in the long run, help to reveal the therapeutic potential of activating TERT in cardiovascular diseases.

## 2. Role of Telomerase and TERT in the Heart

In TERC-deficient mice, which lack Telomerase activity, telomeres are shortened at a rate of 3000–5000 base pairs per generation [10,11,12]. Phenotypic changes in those mice including their heart occur after 3 to 6 generations depending on the genetic background [11,12]. An in-depth analysis of fifth generation (G5) TERC−/− mice in comparison to G2 animals revealed significant shorter telomeres and an increase in p53 expression. Upregulation of p53 in the G5 TERC−/− mice has been linked to increased cardiac myocyte apoptosis, decreased proliferation and cellular hypertrophy. Those alterations led to thinning of the ventricle wall, left ventricular dilatation and impaired cardiac performance [13]. Thus, cardiac dysfunction occurs in G5 TERC−/− mice. An earlier study from the same group already demonstrated that increased proliferation in the heart is linked to a subpopulation of cells showing a colocalization of Ki67, a proliferation marker, with TERT [14]. The authors proposed that those cells could possibly compensate for the ventricular dysfunction.

In humans, telomere shortening and loss of Telomerase activity occurs throughout life [15,16], and amongst various alterations, these changes in the elderly were associated with cardiac dysfunction [13,17,18].

## 3. Role of Telomerase and TERT in Myocardial Infarction and Heart Failure

Telomeres shorten throughout a lifetime and Telomerase activity is decreased in all cells. As this reduced activity is linked to cardiac dysfunction, it was tempting to generate cardiac myocyte-transgenic TERT mice to investigate the changes in the heart. In these animals, telomere shortening in the heart was suppressed. Moreover, ischemic injury after coronary artery occlusion was reduced in the TERT transgenic mice. One underlying mechanism was the anti-apoptotic property of TERT, since apoptosis was inhibited *ex vivo* and *in vivo* [19]. This anti-apoptotic effect of TERT is in line with earlier studies demonstrating that Telomerase promotes cell survival [20,21]. The study of Oh *et al.* [19] provided a first hint for a protective role of TERT in myocardial infarction (Figure 1B). Since the heart consists of several cell types, which probably are all required for regeneration, it was of great interest to identify the cellular population responsible for regeneration/reduced degeneration of myocardial tissue after injury in the adult mouse heart. Therefore, Richardson *et al.* [9] used an mTERT-Green fluorescent protein (GFP)-expressing mouse, in which expression of the transgene is driven by its native promoter [22]. Detectable TERT expression and Telomerase activity were found in adult cardiomyocytes, endothelial cells and fibroblasts by co-staining with cell type specific markers [9] (Figure 1A). The expression of mTERT-GFP decreased with age, which may explain the reduction of Telomerase activity in the myocardium and the increased vulnerability of the heart in the elderly. In response to cryoinjury, the mTERT-GFP mice showed a significant increase in TERT-GFP expressing cells between the injury zone and the surrounding area, which could be interpreted as an indicator for cell proliferation. Those cells were positive for endothelial, fibroblast and cardiac stem cell markers. This study suggests that re-expression of TERT after injury is one important mechanism in mice and possibly also in humans to cope with the reduced functionality after insult. A direct involvement of TERT in regeneration after heart injury was demonstrated in zebrafish, where a strong regenerative capacity had previously been demonstrated [23]. Cryoinjury destroying about 20% of the organ led to a rapid upregulation of telomerase activity and complete regeneration of the heart within 60 days. However, TERT-deficient animals, which, without injury, do not display a heart phenotype, were characterized by incomplete resolution of the initial scar-like fibrotic tissue and a long-term reduction in ventricular function. This impaired regeneration was attributed to reduced cardiomyocyte proliferation, an increase in DNA-damage and the induction of cellular senescence. Interestingly, mildly elevated DNA damage was already observed without injury in these animals, indicating that TERT has protective functions also under homeostatic conditions [24]. Taken together, re-expression of TERT in cardiac myocytes could have therapeutic potential. Following this concept, a recent study used an adeno-associated virus of serotype 9 (AAV9) for TERT re-expression specifically in cardiac myocytes to determine the therapeutic potential in a mouse model of myocardial infarction induced by coronary artery ligation. The main cause of death after myocardial infarction in the FVB/N mice used in this study is the development of heart failure. In the absence of myocardial infarction, the AAV9-TERT treatment did not alter heart morphology in adult mice within 9–10 weeks. Treatment with AAV9-TERT significantly reduced mortality after myocardial infarction and preserved the ejection fraction of the left ventricle (Figure 1B). The infarct and fibrotic scar sizes were smaller in AAV9-TERT-treated mice compared to AAV9-treated mice. Finally, the higher survival rate of the mice after myocardial infarction was accompanied by increased cardiac myocyte proliferation [25]. While several sources of newly cycling cardiac myocytes had been proposed previously [26], the study by Bär *et al.* [25] did not reveal their origin. However, it has been suggested that cardiac injury stimulates pre-existing cardiac myocytes to proliferate [27].

Taking these results regarding the role of Telomerase and TERT in the heart together, it seems to be reasonable to develop new therapeutic strategies based on Telomerase activation to improve the outcome after myocardial infarction and to potentially treat heart failure.

## 4. Physical Exercise, Telomerase and TERT in the Heart

Regular physical activity is associated with a reduced risk for cardiovascular diseases. An improvement in exercise capacity and endothelial function has also been found in patients with coronary artery disease and chronic heart failure [28,29] indicating that physical exercise could be beneficial in these diseases. Several parameters have been associated with regular physical exercise, like improved body weight, blood pressure and a reduction in inflammatory markers. As Telomerase and physical exercise both have a positive impact on the heart, it could be hypothesized that Telomerase plays a role in the protective effects of voluntary exercise. Therefore, myocardial expression of TERT as well as telomere repeat factor 2 (TRF2), which forms the telomere protecting T-loop at the end of the chromosomes [30], was measured in mice after voluntary running for 3 weeks to 6 months. TERT and TRF2 mRNA and protein levels were increased in the heart compared to sedentary control animals (Figure 1A) [7]. Of note, statin treatment—in our day, state of the art medication for patients with cardiovascular diseases—led to an increase in Telomerase activity and TRF2 in human endothelial cells and circulating endothelial progenitor cells [6,31], demonstrating the relevance of Telomerase and TRF2 also in humans. Moreover, myocardial cell apoptosis in human heart failure was linked to down-regulation of TRF2 [32].

Important signs for apoptosis and cell-cycle arrest are the upregulation of the proteins p53 and p16. Voluntary running reduced p53 and p16 protein levels in the heart and as a consequence, basal as well as doxorubicin induced cardiac apoptosis was significantly reduced by voluntary running in mice. Interestingly, all the effects of voluntary running were completely dependent on TERT (Figure 1B), because in TERT-deficient mice, TRF2 is not upregulated, p16 and p53 are not downregulated and no apoptosis protection by voluntary running could be observed [7]. Thus, Telomerase and TERT are absolutely required for the beneficial effects of physical exercise in mice and in humans.

## 5. Role of Telomerase and TERT in the Vascular System

Telomerase activity has been detected in the endothelial and smooth muscle cells of the vessels. Furthermore, it has been demonstrated that coronary endothelial cells from patients with coronary artery disease have shorter telomeres than age- and gender-matched healthy controls [33], indicating loss of Telomerase activity with age and in cardiovascular diseases. Moreover, telomere shortening was more pronounced in endothelial cells from atherosclerotic lesions than from non-diseased areas [33]. This telomere shortening could be caused by oxidative stress [34], which occurs in atherosclerosis-prone regions characterized by disturbed blood flow [35,36] and is increased in many cardiovascular diseases [37]. Interestingly, oxidative stress also induces premature senescence and apoptosis in endothelial cells [38,39], such that telomere shortening could be a consequence of compensatory proliferation required to replace dead cells. With respect to senescence, telomere shortening is discussed as one hallmark of cellular senescence [40] and senescent cells are believed to contribute to tissue dysfunction [41], meaning one could speculate that, already beginning in subclinical stages of atherosclerosis, high hemodynamic stress and/or disturbed blood flow could induce senescence, reduce Telomerase activity and shorten telomeres in the endothelium.

Indeed, Minamino *et al.* observed increased senescence-associated beta Galactosidase activity—a sign of a senescence-associated phenotype—in arterial endothelial cells of human atherosclerotic plaques [42]. Further *ex vivo* investigations demonstrated shorter telomeres, reduced Telomerase activity [42] and decreased expression and activity of endothelial nitric oxide synthase (eNOS) [43,44] in old endothelial cells, all signs for a dysfunctional endothelium. Re-expression of TERT can reverse all these parameters [44]. On the other hand, Telomerase activity can be increased through exogenously adding nitric oxide to old endothelial cells and thereby delay the onset of senescence [45]. Thus, Telomerase and eNOS mutually reinforce their activities in the endothelium. This is underscored by several studies investigating the posttranslational regulation of TERT and Telomerase activity in endothelial cells. An upstream regulator of eNOS is the protein kinase B/Akt1. Akt1 phosphorylates eNOS on serine 1179 in humans, which leads to an increase in eNOS activity [46]. The same kinase has been demonstrated to be required for phosphorylation of TERT on serine 823 in humans leading to enhanced Telomerase activity [6,47,48]. Moreover, it was shown that TERT is in a complex with Akt1 and that nuclear import of TERT is also dependent on Akt1 phosphorylation at serine 227 in humans [48,49].

On the transcriptional level, several transcription factors have been shown to bind to the TERT promoter. The most comprehensive and detailed analysis of the TERT promoter was performed in human tumor cell lines and primary keratinocytes by Kang *et al.* [50]. Interestingly, a new transcription factor was found to be essential for TERT expression and Telomerase activity—Grainyhead like 2 (GRHL2) [50,51]. GRHL2 belongs to a family of transcription factors, which includes as closest relatives GRHL1 and GRHL3 [52]. All of them share an identical consensus binding sequence on DNA [53]. We recently demonstrated that the two long isoforms of GRHL3 inhibit apoptosis, induce migration and angiogenesis of endothelial cells in an eNOS-dependent manner [54,55,56]. All of those are signs for functional endothelial cells and are disturbed in senescent endothelial cells. Moreover, GRHL3 is able to upregulate Akt2 mRNA expression [56]. Akt2 is described as a master regulator of Akt1 activity [57]. Since TERT and Telomerase activity are necessary for apoptosis prevention and senescence inhibition in the endothelium, it is tempting to speculate that GRHL3 is one important transcription factor regulating TERT on the transcriptional level or indirectly by inducing eNOS activation in the endothelium.

Another link between eNOS and TERT in the vasculature has been described in an exercise model in mice. Voluntary running increased TERT mRNA, protein levels and Telomerase activity not only in the heart as described above, but also in the vessels. Those mice show a marked reduction in lipopolysaccharide-induced aortic endothelial cell apoptosis. Moreover, these studies, in which also TERT and eNOS deficient mice were used, revealed that both enzymes synergize to confer endothelial stress resistance after physical activity [8]. Another *in vivo* hint that Akt1 is required for TERT and Telomerase activity in the vessels comes from a study which uses pioglitazone—a Peroxisome proliferator-activated receptor-γ (PPAR-γ) agonist. Pioglitazone is used in the treatment of type 2 diabetes. However, increasing evidence suggests that it also improves vascular functions and prevents atherosclerosis progression [58]. Treatment of mice with pioglitazone for 4 weeks resulted in increased aortic Telomerase activity and phosphorylation of Akt1. Moreover, lipopolysaccharide-induced aortic endothelial apoptosis was dramatically inhibited in pioglitazone treated mice. This inhibitory effect was completely blunted in TERT deficient littermates. Of note, phosphorylation of Akt1 by pioglitazone was not inhibited in TERT deficient mice, indicating also *in vivo* that Akt1 activation is upstream of TERT, but TERT is needed for the anti-apoptotic effect in the endothelium and thus for vascular functionality [59]. In contrast to endothelial cells, pioglitazone as well as overexpression of PPAR-γ blocked mitogen-induced upregulation of TERT protein levels and Telomerase activity in vascular smooth muscle cells. Moreover, a three-day treatment of mice with pioglitazone in a wire injury model leading to removal of the endothelial cell layer of the femoral artery resulted in reduced Telomerase activity in this artery [60]. This finding is probably due to the inhibition of Telomerase activity in the vascular smooth muscle cells, which are the predominant cell type in this short-term injury model after removal of the endothelium. Thus, one could suggest that Telomerase activity is required for vascular smooth muscle cell proliferation. This hypothesis is further supported by the finding that inhibition of telomerase diminishes growth of vascular smooth muscle cells [5].

Recently, a novel role for TERT and Telomerase activity has been described in the vascular system. The vasoactive peptide Angiotensin 1-7, known to increase endothelium-dependent nitric oxide production, has been demonstrated to increase TERT expression and Telomerase activity [61]. The authors of this work propose that the activation of Telomerase is upstream of the increase in NO production. However, this is not proven in this study and from the literature it is only clear that eNOS and TERT have synergistic effects and activate each other. Probably the common mediator of both is the activation of Akt1 as discussed above.

One important issue from the clinical point of view is that medications effectively used in the therapy of cardiovascular diseases have been demonstrated to enhance not only eNOS, but also Telomerase activity and thus delay endothelial cell senescence, leading to a more functional endothelium. One of the most important examples, statins, HMG-CoA reductase inhibitors, a class of lipid-lowering medications, will be discussed here. Statins have several pleiotropic effects. Two of them important in the context of this review are the activation of Akt1 and eNOS. Thus, it is not surprising that statins can increase Telomerase activity and delay senescence of endothelial as well as endothelial progenitor cells [6,62,63].

Interestingly, statins also have an influence on leukocytes. A cross-sectional study of 230 subjects revealed higher telomerase activity and longer telomeres in peripheral blood mononuclear cells, when the subjects were on statin treatment. Furthermore, the age-related decrease in telomere length was reduced by the treatment [64]. A more specific analysis of human T-lymphocytes showed that atorvastatin in pharmacologically relevant doses led to a transient increase in telomerase activity in T-cells. This effect, which could be blocked by inhibitors of Akt and Phosphatidylinositol-4,5-Bisphosphate 3 (PI3)-Kinase, was more pronounced in the CD4-positive (CD4^+^) than in the CD8-positive (CD8^+^) T-cell subset. The upregulation of telomerase activity in CD4^+^ T-cells was accompanied by a moderate increase in the proliferation rate, which was again dependent on Akt. Furthermore, the induction of telomerase activity and cell proliferation was abrogated by coincubation with increasing concentrations of cholesterol, demonstrating that the statin effects were mediated through its effects on cholesterol metabolism. A direct effect of TERT on T-cell proliferation and telomerase activity was demonstrated in mice. Splenocytes from heterozygous and homozygous TERT-deficient animals showed a gene dose dependent reduction in proliferation over four weeks and the atorvastatin-induced upregulation of telomerase activity was completely absent in the knockout situation. This was not a bystander effect of telomere shortening, as the mean telomere length in the first generation TERT knockout mice was identical to wildtype animals [65]. A subset of CD4^+^ T-cells, which in addition express CD25 and the Forkhead Protein 3 (FoxP3), represent natural regulatory T-cells (T_regs_). They are able to suppress several immune cells including Th1-cells and as such could be inhibitors of atherosclerosis, as Th1 cells are the majority of pathogenic T-cells in atherosclerosis and contribute to multiple proatherogenic processes. Interestingly, atorvastatin is capable to induce natural T_regs_ from peripheral CD4^+^ T-cells and can enhance their functional suppressive properties [66]. Given the effect of statins on telomerase activity and the role of TERT in proliferation of CD4^+^ cells suggests that TERT might also have a function in the generation of an anti-inflammatory T-cell response in atherosclerosis via upregulation of T_reg_-numbers.

Surprisingly, a study using mice deficient in the second subunit of telomerase, the RNA component TERC, demonstrated an atheroprotective effect upon loss of functional telomerase [67]. In this study, fourth generation (G4) TERC-knockout mice on an apolipoprotein E-deficient background fed a high-fat diet showed a reduction in atherosclerotic lesion area and intimal thickening compared to TERC-proficient animals. The authors suspected that the reduction in severity of the disease might be due to impaired proliferative capacity of leukocytes, including cell types promoting atherosclerosis progression. However, the data should be considered with caution, because these late generation TERC-deficient mice are characterized by a number of other defects, including e.g. an increased frequency of chromosomal end-to-end fusions, aneuploidy and impaired stress-responses, which make the interpretation of complex phenotypes like atherosclerosis extremely difficult.

**Figure 2:**
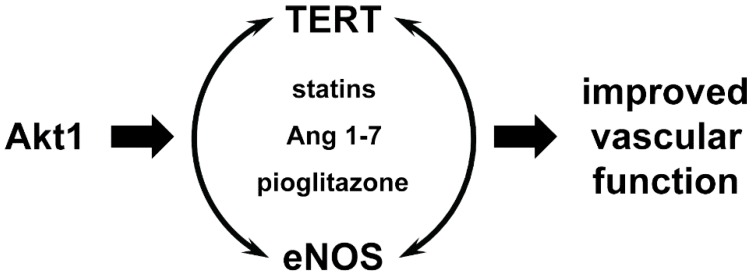
Interconnection between TERT and eNOS. TERT and eNOS mutually reinforce their activities in the endothelium. One common upstream regulator is Akt1. Statins, Angiotensin 1-7 (Ang1-7) and pioglitazone increase not only NO production, but also Telomerase activity.

Taking all of the findings in the vascular system together, it is proven that TERT protein and Telomerase activity are present in the vessel and loss of the enzyme results in vascular dysfunction. eNOS and TERT mutually reinforce each other and are dependent on each other (Figure 2). Statins, which improve endothelial function, were thought to act on eNOS; however, recent evidence supports the notion that they also act on TERT and Telomerase activity (Figure 2), not only in the vascular wall, but also in cells of the immune system. Finally, TERT deficiency and thus loss of Telomerase activity result in diminished vascular protection and increased senescence.

## 6. Extranuclear Functions of TERT in the Cardiovascular System

Besides the important role of TERT in the nucleus, there is accumulating evidence that TERT is not only localized in this organelle, but also in mitochondria of several cell types including cardiovascular cells [68,69,70,71]. Several studies have shown that TERT is localized in the inner mitochondrial membrane and associated with mitochondrial DNA as well as mitochondrial tRNA [71,72]. However, the function of TERT in the mitochondria is still not fully understood.

Intriguingly, TERT improves oxygen consumption in vascular cells and this depends on its catalytic activity [71]. Optimal respiratory chain function requires proper assembly of the respective complexes in the inner mitochondrial membrane. Thirteen of the subunits of these complexes are expressed from mitochondrial DNA, whereas the majority is encoded by the nuclear genome. Thus, complex assembly depends on a tightly controlled coordination between the expression of the nuclear and mitochondrial genes encoding these proteins. It has been demonstrated that TERT interacts with RMRP, the RNA component of a mitochondrial RNA processing ribonuclease [73]. This complex has an RNA-dependent RNA polymerase (RdRP) activity, which depends on catalytically active TERT. It produces double stranded RNAs serving as precursors for small interfering RNAs [73], which can alter gene expression programs. Interestingly, one important regulator of TERT in the nucleus, Akt1, has also been found in the mitochondria of endothelial cells, where it is inhibited by stimuli known to reduce nuclear Telomerase activity like oxidative stress [74]. One could speculate that TERT is activated by Akt1 in the mitochondria and Telomerase activity in conjunction with RMRP is involved in the control of gene expression, possibly also of genes encoding respiratory chain complexes. Moreover, oxidative stress in non-proliferating cells induces nuclear export of TERT [70,75] and an increase of TERT in the mitochondria has been observed under those conditions [70]. Therefore, one could hypothesize that TERT will be transported to organelles within the cell where Telomerase or RdRP activity is needed to guarantee cell survival. At a point of no return for the cell, a reduction in TERT protein levels is observed [74,76].

Taking these findings together, an activity of Telomerase seems to be present within mitochondria. Mitochondrial TERT improves mitochondrial parameters in the cardiovascular system. Thus, it is important to understand whether nuclear or mitochondrial TERT, or both, are protective in the cardiovascular system in order to develop therapeutic approaches aimed at enhancing TERT function.
